# AUF1 promotes stemness in human mammary epithelial cells through stabilization of the EMT transcription factors TWIST1 and SNAIL1

**DOI:** 10.1038/s41389-020-00255-1

**Published:** 2020-08-05

**Authors:** Manar M. AlAhmari, Huda H. Al-Khalaf, Falah H. Al-Mohanna, Hazem Ghebeh, Abdelilah Aboussekhra

**Affiliations:** 1 Department of Molecular Oncology, King Faisal Specialist Hospital and Research Centre, MBC#03, Riyadh, 11211 Saudi Arabia; 2 The National Center for Biotechnology, King Abdulaziz City for Science and Technology, Riyadh, 11461 Saudi Arabia; 3 KACST-BWH/Harvard Center of Excellence for Biomedicine, Joint Centers of Excellence Program, King Abdulaziz City for Science and Technology (KACST), Riyadh, 11461 Saudi Arabia; 4 Department of Comparative Medicine, King Faisal Specialist Hospital and Research Center, Riyadh, 11211 Saudi Arabia; 5 Stem Cell & Tissue Re-Engineering Program, King Faisal Specialist Hospital and Research Centre, MBC#03, Riyadh, 11211 Saudi Arabia

**Keywords:** Breast cancer, Cancer stem cells

## Abstract

The AU-rich element RNA-binding protein 1 (AUF1) is an RNA-binding protein, which can both stabilize and destabilize the transcripts of several cancer-related genes. Since epithelial-to-mesenchymal transition (EMT) and the acquisition of cancer stem cell traits are important for cancer onset and progression, we sought to determine the role of AUF1 in these two important processes. We have shown that AUF1 induces EMT and stemness in breast epithelial cells via stabilization of the *SNAIL1* and *TWIST1* mRNAs, and their consequent upregulation. Indeed, AUF1 binds the transcripts of these two genes at their 3′UTR and reduces their turnover. Ectopic expression of AUF1 also promoted stemness in mammary epithelial cells, and thereby increased the proportion of cancer stem cells. Importantly, breast cancer cells that ectopically express AUF1 were more efficient in forming orthotopic tumor xenografts in nude mice than their corresponding controls with limiting cell inocula. On the other hand, AUF1 downregulation with specific siRNA inhibited EMT and reduced the stemness features in breast cancer cells. Moreover, AUF1 knockdown sensitized breast cancer cells to the killing effect of cisplatin. Together, these findings provide clear evidence that AUF1 is an important inducer of the EMT process through stabilization of *SNAIL1* and *TWIST1* and the consequent promotion of breast cancer stem cells. Thereby, AUF1 targeted molecules could constitute efficient therapeutics for breast cancer patients.

## Introduction

Breast cancer (BC) is the most common cancer in females worldwide, with nearly 25% of all new cancer cases and 15% of all cancer deaths^1^. Considerable advances have been made in the treatment of BC, including local interventions (surgery/radiotherapy) and systemic therapies such as chemo/hormonal and targeted treatments^2,3^. However, BC recurrence, chemotherapy resistance, and metastasis remain major problems. Recent evidences suggest that BC recurrence and chemotherapy resistance is mainly due to the presence of a small subpopulation of cells known as BC stem cells (BCSCs)^4,5^. These cells possess stem cell-like features, such as self-renewal and differentiation capacities, which can produce different lineages of cancer cells^5,6^. Furthermore, gaining of stemness traits involves the epithelial-to-mesenchymal transition (EMT) process^7,8^. The activation of such process is under the control of several transcription factors, such as ZEB1 and TWIST1 (refs ^9–11^).

The control of mRNA stability is one of the most important post-transcriptional mechanism of gene expression regulation^10^. This process is exerted via the binding of RNA-binding proteins (RBPs) to adenylate–uridylate-rich elements (ARE) within the 3′ untranslated region (3′UTR) of mRNAs^12,13^. The AU-binding factor (AUF1), also called heterogeneous nuclear ribonucleoprotein D (hnRNPD), is one of the best characterized RBPs, which recognizes ARE sequences within different transcripts and controls their stability^14^. Alternative splicing of the AUF1 transcript produces four isoforms, which code for p37, p40, p42, and p45, and all have RNA-binding ability with different affinities^15^. The p37 isoform showed the highest affinity for RNA targets as well as pro-carcinogenic effects in transgenic mice^15–18^. The well-known function of AUF1 is the destabilization of mRNAs by forming complexes with other proteins. However, recent studies reported an mRNA-stabilizing role of AUF1 (refs ^14,19^). Importantly, other studies have shown a considerable increase in the AUF1 expression in tumor cells^20^. Furthermore, AUF1 stabilized the mRNA of the pro-EMT transcription factor ZEB1 in osteosarcoma and thyroid cancer cells^21,22^. These data prompted us to ask whether AUF1 upregulation could promote EMT as well as stemness in mammary epithelial cells. Indeed, we have shown that AUF1 induces EMT and stemness in breast epithelial cells via stabilization of the *SNAIL1* and *TWIST1* mRNAs, and their consequent upregulation. Our data propose a new strategy for BC treatment, whereby AUF1 inhibition could suppress EMT/stemness, which should promote chemosensitivity and/or prevent cancer relapse.

## Results

### Ectopic expression of AUF1 promotes the EMT process in breast epithelial cells

**Fig. 1:**
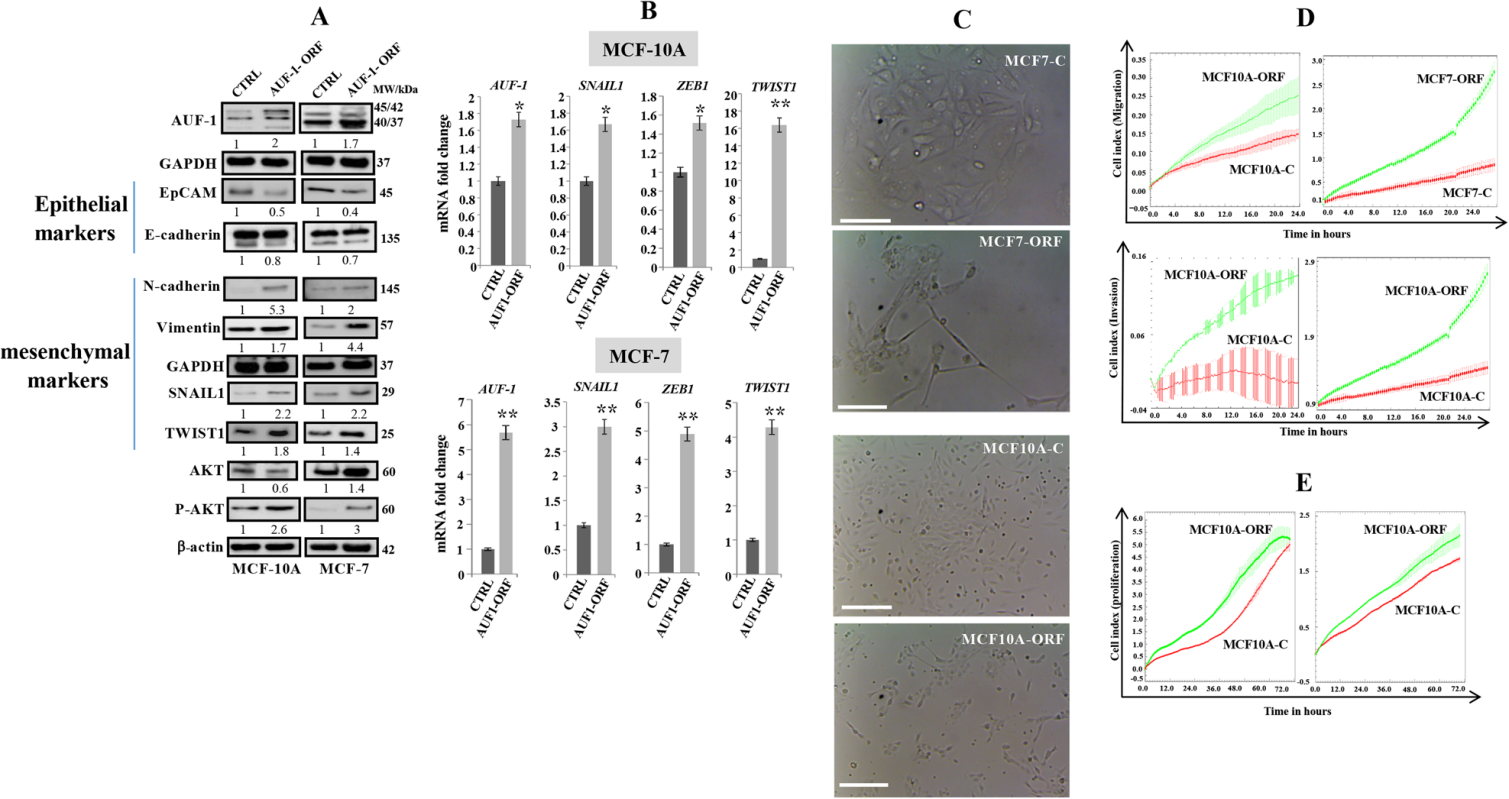
Ectopic expression of AUF1 induces EMT in mammary epithelial cells. **a** MCF10A and MCF7 cells were infected with lentivirus-based vectors either empty (MCF10A-C) (MCF7-C) or bearing p37^AUF1-ORF^ (MCF10A-ORF) (MCF7-ORF). Whole-cell lysates were prepared from these cells and were used for immunoblotting using antibodies against the indicated proteins. The numbers bellow the bands represent fold change relative to the corresponding control after correction against the internal controls β-actin or GAPDH. The levels of phosphorylated proteins were normalized against the total amount of their relative non-phosphorylated forms. **b** Total RNA was purified, and then amplified by qRT-PCR. The relative mRNA expression levels were normalized against GAPDH. Error bars represent means ± SD (*P < 0.05, **P < 0.01). **c** Representative images of cells. Scale bar, 50 μm. **d** Exponentially growing cells were added in SFM to the upper wells of the CIM plates either separated by a matrigel basement membrane matrix (Invasion) or without (Migration), and the migration/invasion were assessed for 24 h using the RTCA-DP xCELLigence System. **e** Exponentially growing cells were added in complete medium to the wells of an electronic microtiter plate (E-plate). The proliferation rate was measured for a period of 72 h.

We started the present study by investigating the potential implication of AUF1 in promoting breast carcinogenesis, through inducing EMT in epithelial cells. To this end, we have first ectopically expressed AUF1 in the non-carcinogenic breast epithelial cells (MCF10A) and the luminal breast cancer cells (MCF7). These cells were infected with lentivirus-based vectors either empty (MCF10A-C) (MCF7-C) or bearing p37^AUF1-ORF^ (MCF10A-ORF) (MCF7-ORF). Whole-cell lysates were prepared from these cells and the levels of AUF1 and the main EMT markers were assessed by immunoblotting utilizing specific antibodies, while GAPDH and β-actin were used as internal controls. Figure 1a shows that p37^AUF1-ORF^ increased the level of the four AUF1 isoforms. This could be mediated indirectly through the positive IL-6/STAT3 feedback loop^19^. Concomitantly, the level of the major mesenchymal markers (N-cadherin, Vimentin, SNAIL1, and TWIST1) were also increased, whereas the levels of the epithelial markers EpCAM and E-cadherin were reduced in both cell lines (Fig. 1a). These results were confirmed at the mRNA level by quantitative reverse transcription PCR (qRT-PCR). Indeed, ectopic expression of AUF1 significantly increased the mRNA level of the three EMT-TFs (*SNAIL1*, *ZEB1*, and *TWIST1*) in MCF10A-ORF and MCF7-ORF cells compared to their respective controls (Fig. 1b). In addition to these molecular changes, we have also observed morphological changes in some MCF7 cells expressing p37^AUF1-ORF^, while these phenotypic changes were not obvious for AUF1-expressing MCF10A cells (Fig. 1c). In order to confirm the effect of AUF1 in inducing EMT, we assessed the migration/invasion and proliferation abilities of MCF10A-ORF/MCF10A-C and MCF7-ORF/MCF7-C cells. Thereby, cells were seeded in the upper wells of the CIM plate in the presence of matrigel basement membrane matrix (invasion) or without (migration), while the lower wells contained the appropriate complete medium. Cellular invasion and migration abilities were assessed using the RTCA-DP xCELLigence system for 24 h. Figure 1d shows that AUF1 upregulation potently increased the migration and invasion capacities of MCF10A and MCF7 cells as compared to control cells. Next, MCF10A-ORF/MCF10A-C and MCF7-ORF/MCF7-C cells were added in their appropriate complete medium to the wells of an E-plate and the proliferation rates of such cells were assessed using the RTCA-DP xCELLigence system for 72 h. As expected, MCF10A-ORF and MCF7-ORF cells exhibited higher proliferation rate relative to their respective controls (Fig. 1e). To explore the molecular mechanism underlying the increase in the migration/invasion and proliferation capacities of MCF10A and MCF7 cells expressing p37^AUF1-ORF^, we investigated the possible involvement of the pro-invasive/migratory and proliferative protein kinase AKT by immunoblotting. Interestingly, the ectopic expression of AUF1 increased the level of the active/phosphorylated form of AKT (Fig. 1a). These findings indicate that AUF1 upregulation promotes EMT in mammary epithelial cells.

### AUF1 downregulation inhibits the EMT process in BC cells

**Fig. 2:**
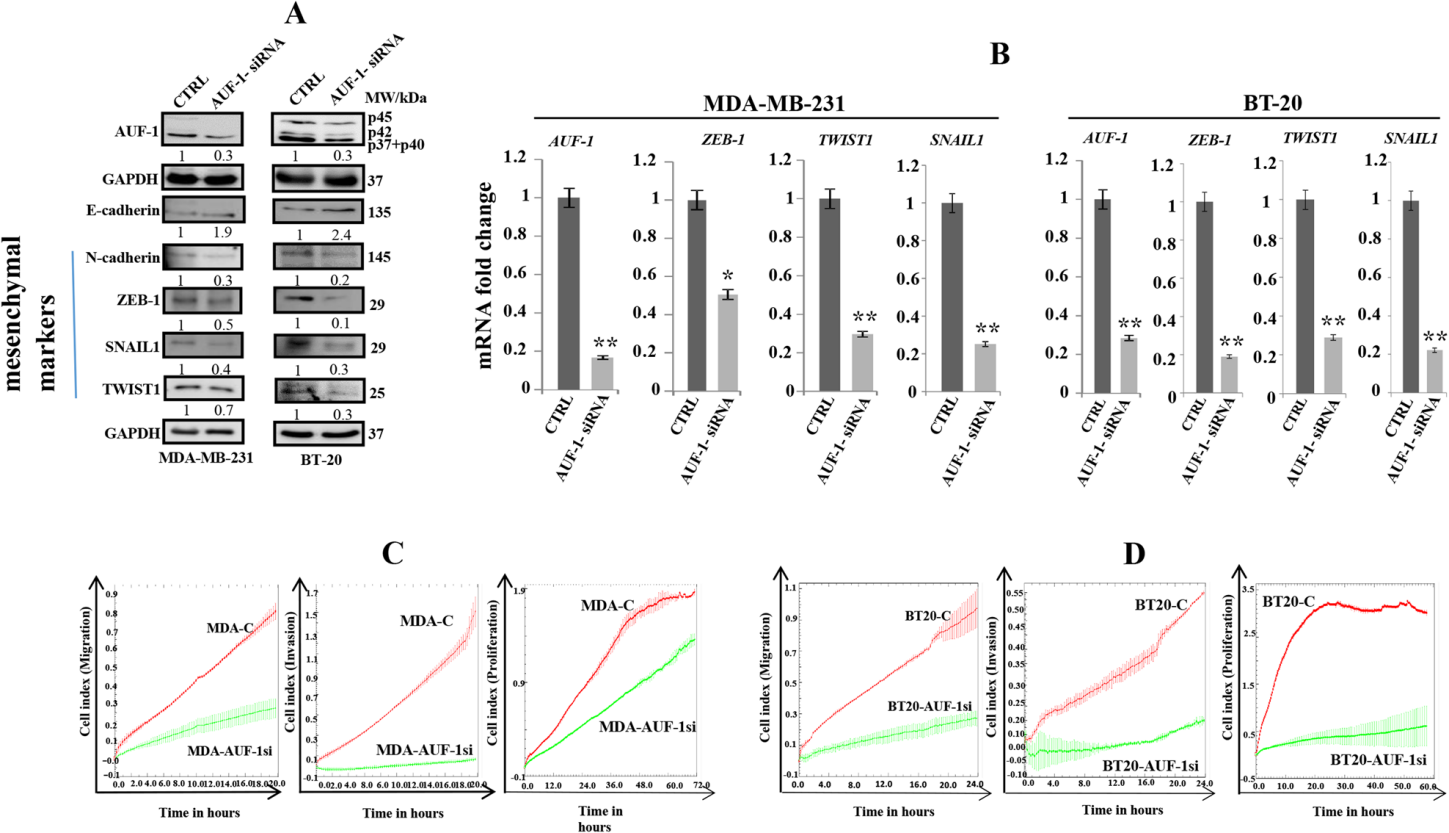
AUF1 downregulation inhibits EMT in TNBC cells. **a** MDA-MB-231 and BT-20 cells were transfected with control or AUF1-siRNA (MDA-AUF1si/MDA-C) or (BT20-AUF1si/BT20-C), respectively. Whole-cell lysates were prepared and were used for immunoblotting analysis using antibodies against the indicated proteins, and GAPDH was used as an internal control. The numbers bellow the bands represent fold change relative to the corresponding control. **b** Total RNA was purified from the indicated cells and used for qRT-PCR. Error bars represent means ± SD (*P < 0.05, **P < 0.01). **c**, **d** Cell proliferation, migration, and invasion abilities were assessed for the indicated periods of time using the RTCA-DP xCELLigence System.

To confirm the role of AUF1 in EMT, we have explored the effect of AUF1 downregulation in reversing the EMT process. Thus, AUF1 was knocked-down using specific siRNA and a scrambled sequence was used as control in two triple-negative breast cancer (TNBC) cell lines: MDA-MB-231 (MDA-AUF1si/MDA-C) and BT-20 (BT20-AUF1si/BT20-C) cells. These two cell lines are known to express higher levels of the mesenchymal markers compared to other BC cell lines^23^. AUF1 knockdown in MDA-MB-231 cells upregulated the epithelial markers EpCAM and E-cadherin (4.9-fold) and suppressed the four major mesenchymal markers SNAIL1, TWIST1, ZEB1, and N-cadherin (Fig. 2a). Similar results were obtained for BT-20 cells (Fig. 2a). Figure 2b shows that AUF1 downregulation significantly reduced the mRNA levels of the EMT-TFs *SNAIL1*, *ZEB1*, and *TWIST1* in MDA-MB-231 and BT-20 cells as compared to their respective controls. These results indicate that AUF1 has an important role in inducing EMT in BC cells. Additionally, following AUF1 downregulation, the migration, and invasion capacities of MDA-MB-231 and BT-20 cells was significantly reduced, suggesting that AUF1 plays a major role in the migratory/invasiveness capacities of BC cells (Fig. 2c, d). Moreover, MDA-AUF1si and BT20-AUF1-si cells exhibited lower proliferation rate compared to their respective controls (Fig. 2c, d). Similar results were obtained when AUF1 was downregulated with a plasmid bearing specific siRNA pSILENCER-*AUF1-*siRNA that targets all four AUF1 isoforms^24^ (Supplementary Fig. S1). Indeed, AUF1 knockdown promoted the mesenchymal-to-epithelial process and inhibited cellular migration/invasion and proliferation capacities (Supplementary Fig. S1). These results indicate that AUF1 downregulation inhibits the EMT process in BC cells.

### AUF1 binds and stabilizes the *TWIST1* and *SNAIL1* mRNAs

**Fig. 3:**
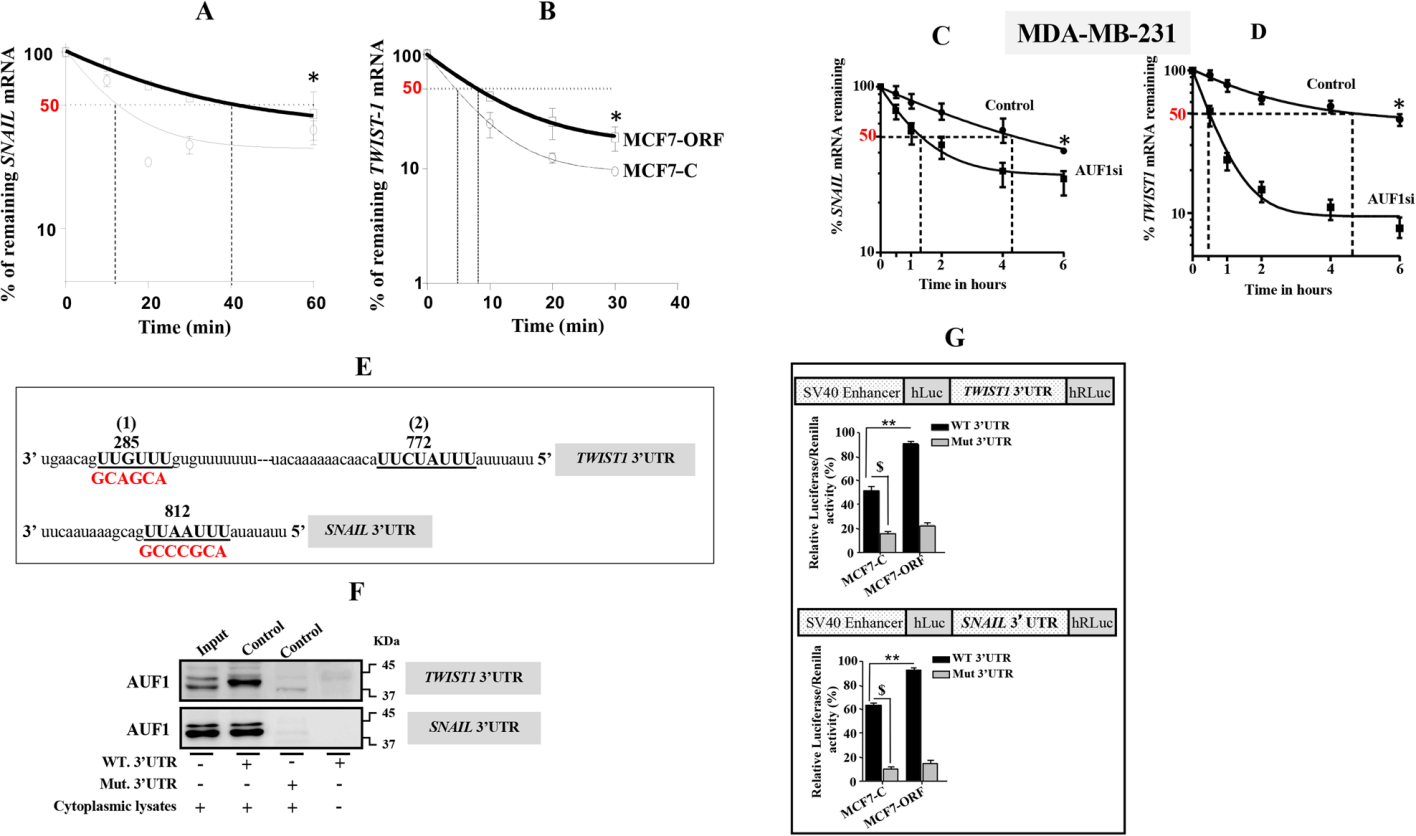
AUF1 stabilizes *SNAIL1* and *TWIST1* mRNAs. **a**–**d** Cells were treated with actinomycin D (5 μg/ml), and then total RNA was extracted at different periods of time, and was subjected to qRT-PCR. Error bars represent means ± SD (*P < 0.05, **P < 0.01). **e** Sequence alignment of the indicated human mRNA 3′UTR showing the WT and the mutated potential AUF1-binding sites. **f** Biotinylated 3′UTR for the indicated mRNAs bearing either WT or mutated sequence of the AUF1-binding site was incubated with cytoplasmic cellular lysate from MDA-MB-231 cells expressing the indicated constructs, and the association of AUF1 with these mRNAs was detected by immunoblotting. **g** MCF7-C and MCF7-ORF cells were stably transfected with the luciferase reporter vector bearing either wild-type *TWIST1* and *SNAIL1* 3′UTR or their mutated sequences as shown in **e**. The reporter activity was assessed at 48 h post-transfection. Data (mean ± SEM, *n* = 4) were presented as % change in reporter activity as compared to control cells (**) or to the mutated 3′UTR ($). **P < 0.002 and ^$^*P* < 2 × 10^–5^.

To elucidate the molecular mechanism that underlies AUF1-dependent upregulation of *SNAIL1* and *TWIST1* and the consequent induction of EMT, we sought to investigate the effect of AUF1 on the stability of their transcripts in cells expressing a high level of AUF1 (MCF7-ORF). To this end, MCF7-ORF/MCF7-C cells were treated with the transcription inhibitor actinomycin D (5 μg/ml), and then were reincubated for different periods of time. Total RNA was purified and amplified with qRT-PCR using specific primers. Figure 3a shows that AUF1 ectopic expression increased the *SNAIL1* mRNA half-life. Indeed, while the *SNAIL1* mRNA half-life reached 40 min in MCF7-ORF cells, it was only 15 min in MCF7-C cells (Fig. 3a). Likewise, AUF1 ectopic expression increased the *TWIST1* mRNA half-life from 5 to 8 min (Fig. 3b). On the other hand, AUF1 downregulation by specific siRNA in MDA-MB-231 cells increased the turnover of both *TWIST1* and *SNAIL1* mRNAs (Fig. 3c, d). These findings indicate that AUF1 stabilizes the *SNAIL1* and *TWIST1* mRNAs.

### AUF1 binds the *TWIST1* and *SNAIL1* mRNAs at their 3′UTR

Next, we sought to explore the molecular mechanism underlying AUF1-dependent positive regulation of the mesenchymal markers TWSIT1 and SNAIL1. Since AUF1 is an RBP, we first searched for AUF1- binding site(s) on the 3′UTR of the *TWIST1* and *SNAIL1* mRNAs, and we have found two different AUF1-binding sites on the *TWIST1* 3′UTR, and one on the *SNAIL1* 3′UTR (Fig. 3e). To prove the binding of AUF1 to the *TWIST1* and *SNAIL1* 3′UTR, biotinylated 3′UTR of each message spanning either the wild type (WT) or the mutated AUF1-binding site were synthesized and incubated with cytoplasmic cellular lysates prepared from MDA-MB-231 cells. The 3′UTR/AUF1 ribonucleoprotein complexes were precipitated and the level of AUF1 was assessed by immunoblotting. Figure 3f shows that AUF1 was associated with the two 3′UTR sequences in control cells. However, when 3′UTRs bearing mutated AUF1-binding sites were used, AUF1 was almost undetectable (Fig. 3f). This shows the binding of AUF1 to the *3*′*UTR* sequences of the *TWIST1* and *SNAIL1* mRNAs in vitro. To confirm this, we investigated the potential contribution of the AUF1-binding sites in the *TWIST1* and *SNAIL1* mRNA 3′UTR on the expression of these genes. To this end, wild-type *TWIST1 and SNAIL1* 3′UTR or the mutated sequences were inserted into a luciferase/Renilla reporter vector and were introduced into MCF7-ORF and MCF7-C cells. The reporter activity fused to the intact sequence of the 3′UTRs was significantly increased in MCF7-ORF as compared to the control cells (Fig. 3g). Interestingly, the activity was strongly reduced by mutating the putative AUF1-binding site within the 3′UTR of both mRNAs (Fig. 3g). This further indicates that the effect of AUF1 is mediated through interaction with its seeding sequence in the *TWIST1* and *SNAIL1* 3′UTRs.

### Ectopic expression of AUF1 induces stemness in mammary epithelial cells

**Fig. 4:**
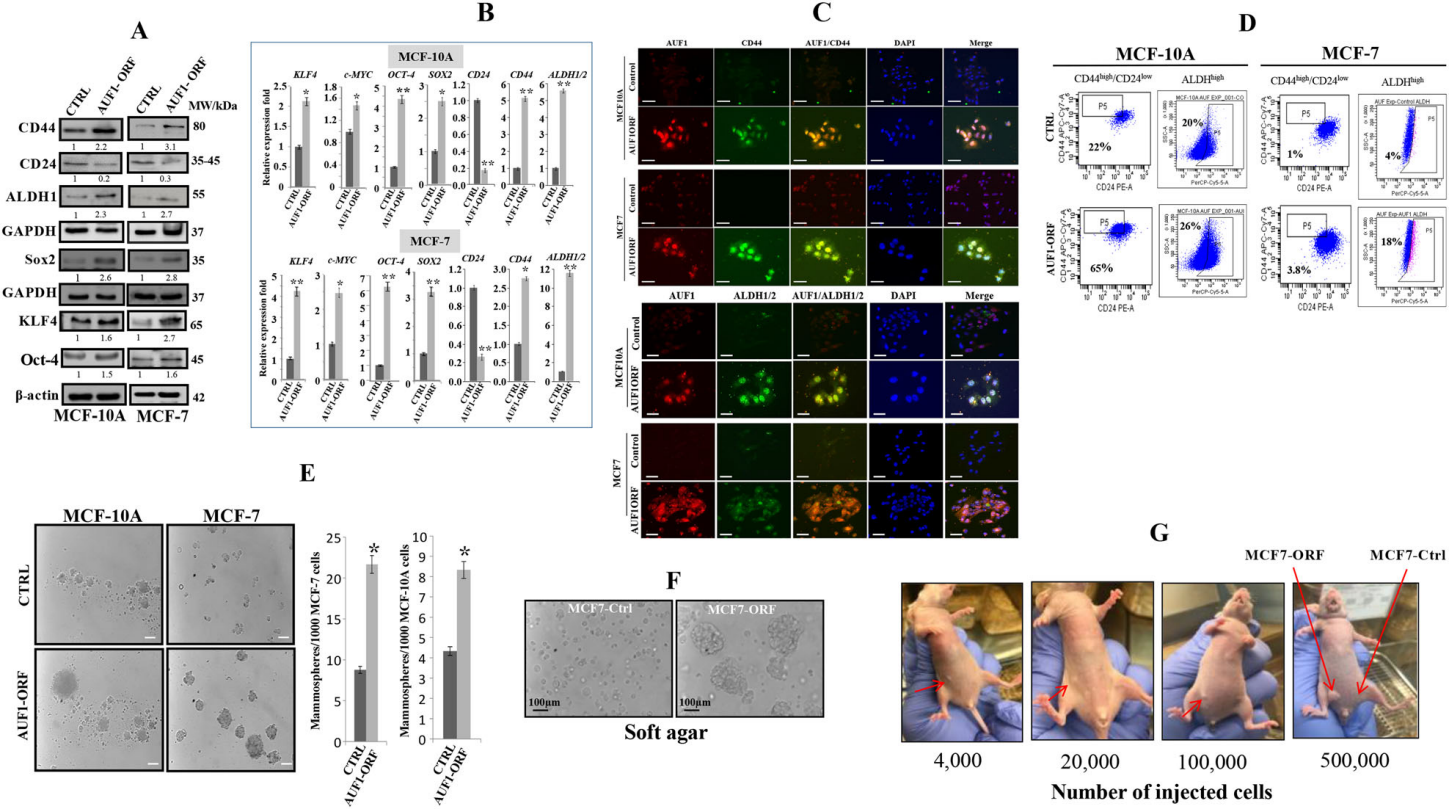
Ectopic expression of AUF1 induces stemness in MCF10A and MCF7 cells. **a** Whole-cell lysates were prepared from the indicated cells and were used for immunoblotting using antibodies against the indicated proteins, and GAPDH and β-actin were used as internal controls. The numbers below the bands represent fold change relative to the corresponding control after correction against the internal controls β-actin or GAPDH. **b** Total RNA was purified from the indicated cells and used for qRT-PCR. Error bars represent means ± SD (*P < 0.05, **P < 0.01). **c** Immunofluorescence assay for the indicated cells using antibodies against the indicated proteins. Scale bars represent 25 µm. **d** FACS analysis of CD44^high^/CD24^low^ and ALDH^high^ stem cell-like subpopulation in the indicated cells. The numbers in the boxes indicate the proportion of cells. **e** Cells (1000) were cultured in ultra-low attachment 96-well plates in the presence of specific stem cell medium. Left panel, representative images of mammospheres. Scale bar, 50 μm. Right panel, graphs depicting number of formed mammospheres. Experiments were performed in triplicate and several times; error bars represent means ± SD (*P < 0.05). **f** Cells were plated in soft agar for colony formation. After 2 weeks colonies were photographed using an inverted microscope. **g** Female nude mice were injected with the indicated number of MCF7-ORF and MCF7-C cells under the right and left nipples, respectively. Five months post-injection, pictures of tumors were taken. Red arrows indicate the grown tumors in mice.

The fact that several studies have shown that the induction of EMT leads to the acquisition of stem cell characteristics^7,8^ prompted us to ask whether AUF1-mediated EMT induces stemness as well. To address this hypothesis, we tested the effect of AUF1 ectopic expression on the protein level of common stem cell and pluripotency markers in MCF10A-ORF/MCF10A-C and MCF7-ORF/MCF7-C cells. Immunoblotting analysis revealed that the ectopic expression of AUF1 resulted in 2–3-fold increase in the stem cell markers CD44 and ALDH1 (Fig. 4a). On the other hand, the CD24 protein was downregulated 5- and 3.3-fold in MCF10A-ORF and MCF7-ORF cells relative to their respective controls (Fig. 4a). In addition, the protein level of the pluripotency markers KLF4, Oct-4, and Sox2 were also higher in MCF10A-ORF and MCF7-ORF cells compared to their respective controls (Fig. 4a). The AUF1-mediated modulation in the expression of the stemness genes *CD24*, *CD44*, *ALDH1*, as well as the pluripotency genes *OCT-4*, *KLF4*, *SOX2*, and *c-MYC* was confirmed at the mRNA level using qRT-PCR (Fig. 4b). To confirm the link between AUF1 and the expression of these stemness-related genes, co-staining immunofluorescence experiments were performed. Figure 4c shows that cells that express a high level of AUF1 stained also positive for CD44 and ALDH1/2. Moreover, the proportion of the two CSC subpopulations: CD44^high^/CD24^low^ and ALDH^high^ was quantified by FACS. Figure 4d shows that the proportion of CD44^high^/CD24^low^ subpopulations in MCF10A-ORF and MCF7-ORF cells increased 3- and 3.8-fold compared to their respective controls. Similarly, FACS analysis revealed that MCF10A-ORF and MCF7-ORF favored the generation of ALDH^high^ subpopulations in comparison to their respective controls (Fig. 4d).

Next, we investigated the implication of AUF1 in mammosphere formation in MCF7 and MCF10A cells. To this end, MCF10A-ORF/MCF10A-C and MCF7-C/MCF7-ORF cells were plated at low density into ultra-low attachment plates in the presence of stem cells culture medium. Cells were incubated for 10 days and the formed mammospheres with diameters of ≥50 μm were counted under the microscope. MCF10A and MCF7 cells ectopically expressing AUF1 demonstrated increase in both size (growth rate) and frequency of spheroids compared to their respective controls (Fig. 4e). Similarly, while control cells did not form colonies (more than 100 μm) in soft agar, MCF7-ORF cells formed big colonies (Fig. 4f). These results indicate that AUF1 promotes (CD44^high^/CD24^low^/ALDH^high^) stemness features in breast epithelial cells and induces the formation of BCSCs.

### AUF1 ectopic expression induces tumorigenicity in vivo

To confirm AUF1-dependent formation of BCSC, we injected MCF7-ORF/MCF7-C cells at different concentrations (4 × 10^3^, 2 × 10^4^, 10^5^, 5 × 10^5^*n* = 3 for each cell concentration) under the right and left nipples of female nude mice, respectively. After 5 months, while control cells did not form tumors, MCF7-ORF cells generated orthotopic tumor xenografts in all injected animals with volumes proportional to the number of cells injected, reaching a volume of 100–150 mm^3^ for those injected with 5 × 10^5^ cells (Fig. 4g). These results indicate that AUF1 promotes the formation of CSCs that are responsible for the growth of orthotopic tumor xenografts.

### AUF1 promotes stemness in mammary epithelial cells in a TWIST1/SNAIL1-dependent manner

**Fig. 5:**
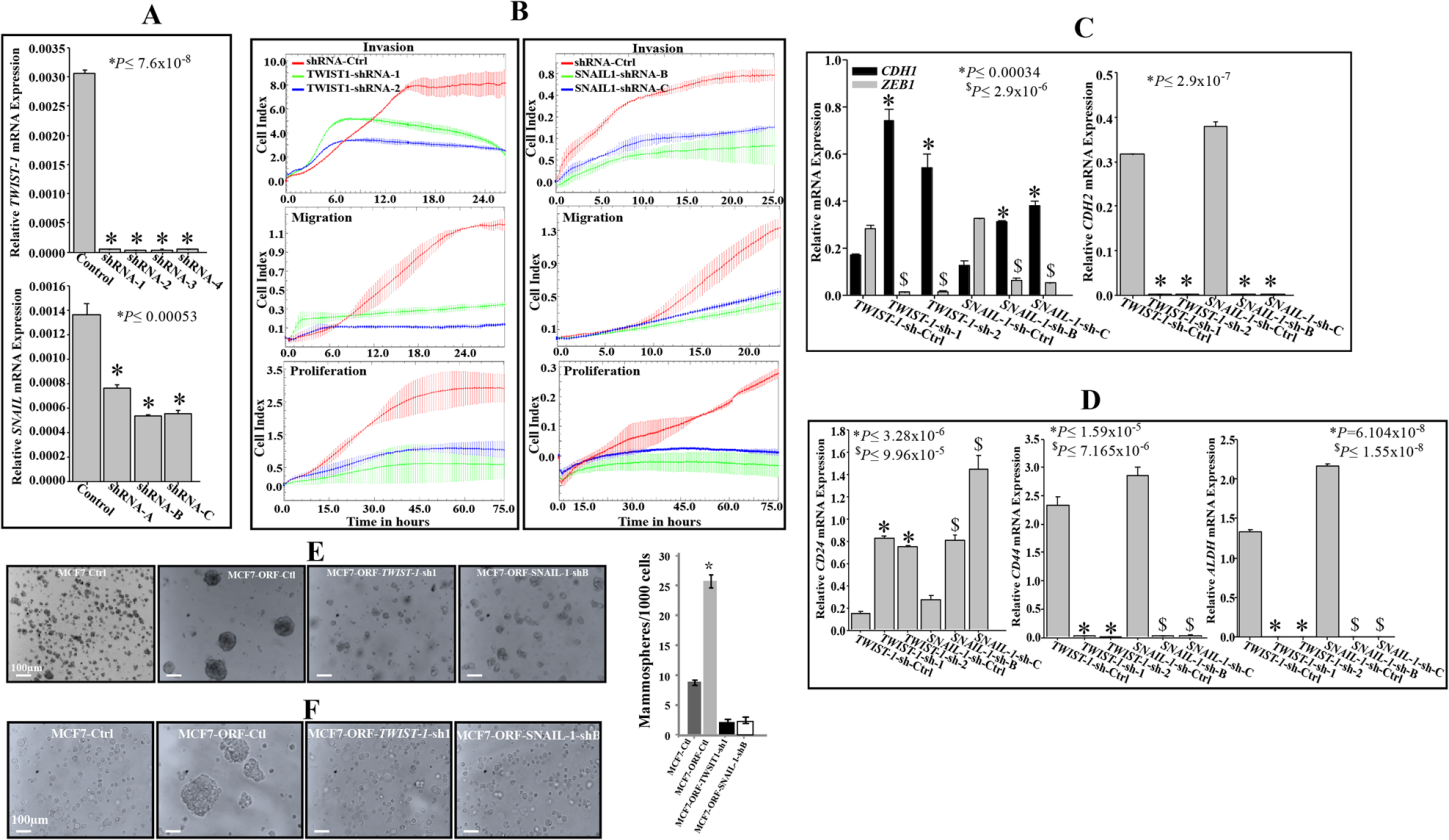
AUF1-depenent induction of stemness is TWIST1-SNAIL1-related. **a** MCF7 cells expressing AUF1-ORF were transfected with plasmids bearing different sequences of either TWIST1-shRNA, SNAIL1-shRNA or a scrambled sequence. After 72 h, cells were harvested and total RNA was purified and used for qRT-PCR. Error bars represent means ± SD. **b** The migration/invasion as well as proliferation capabilities of the indicated cells were assessed using the RTCA-DP xCELLigence system. **c**, **d** Figure legends as in Fig. 4**b**, **e**, **f**. Figure legends as in Fig. 4e, f. Error bars represent means ± SD (*P < 0.05).

To explore the role of TWIST1 and SNAIL1 in AUF1-dependent induction of stemness, we studied the effect of the separate downregulation of these two genes on the expression of the EMT and stem cell markers, in cells ectopically expressing AUF1. Therefore, TWIST1 and SNAIL1 were downregulated using specific shRNA (four different sequences for TWIST1 and three different sequences for SNAIL1) in the MCF7 cells expressing p37^AUF1-ORF^ separately. A scrambled sequence was used as control. Figure 5a shows that all the utilized shRNA sequences knocked-down both *TWIST1* and *SNAIL1* gene. Subsequently, only two shRNA sequences were utilized to assess the migration/invasion and proliferation abilities of these cells. Figure 5b shows that downregulation of TWIST1 or SNAIL1 strongly repressed the migration/invasion as well as the proliferation abilities of these cells, as compared to controls. This indicates that TWIST1 and SNAIL1 are important for AUF1-dependent activation of the migration/invasion and proliferation capacities in MCF7 cells. Interestingly, while downregulation of TWIST1 or SNAIL1 decreased the mRNA levels of the *CDH2* and *ZEB1* genes, they increased the level of the *CDH1* mRNA in cells expressing high level of AUF1 (Fig. 5c). In addition, downregulation of TWIST1 or SNAIL1 decreased the level of the *CD44* and *ALDH1* mRNAs, while it increased the level of the *CD24* mRNA (Fig. 5d). This indicates that the AUF1-dependent modulation in the expression of mammary stem cell-related markers is mediated through TWIST1 and SNAIL1. To confirm this, we investigated the role of these two genes in AUF1-dependent promotion of tumorsphere formation in MCF7 cells. MCF7-Ctl, MCF7-ORF-Ctl as well as MCF7-ORF cells expressing either TWIST1 or SNAL1 shRNAs (MCF7-ORF-TWIST1-sh1, MCF7-ORF-SNAIL1-shB) were plated at low density into ultra-low attachment plates in the presence of stem cells culture medium. Cells were incubated for 10 days and the formed mammospheres with diameters of ≥50 μm were counted under the microscope. While control MCF7 cells ectopically expressing p37^AUF1-ORF^ demonstrated increase in both size (growth rate) as well as frequency of spheroids as compared to their respective controls, TWIST1 or SNAIL1 downregulation reduced the ability of these cells in forming mammospheres (Fig. 5e). Similarly, while control cells did not form colonies in soft agar, MCF7-ORF-Ctl cells formed big colonies; however, no colonies were formed in TWIST1- or SNAIL1-deficient cells (Fig. 5f). This indicates that the AUF1-dependent promotion of stemness in mammalian cells is mediated through TWIST1 and SNAIL1.

### AUF1 knockdown decreases stemness in BC cells

**Fig. 6:**
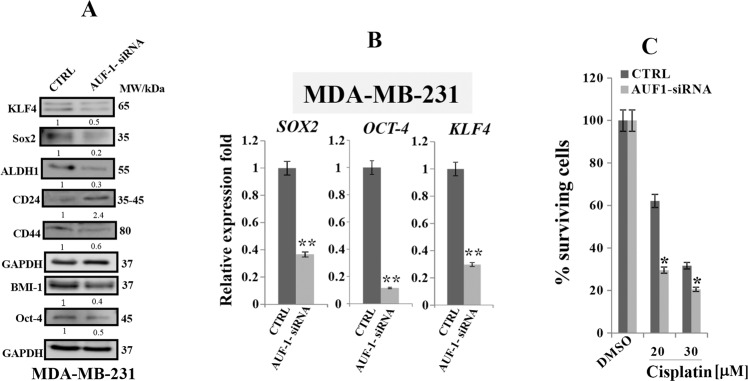
AUF1 downregulation decreases stemness in TNBC cells and enhances their sensitivity to cisplatin. **a** MDA-MB-231 cells were transfected with specific AUF1 siRNA (AUF1-siRNA) or a scrambled sequence (CTRL), and then whole-cell lysates were prepared and were used for immunoblotting using antibodies against the indicated proteins. The numbers bellow the bands represent fold change relative to the corresponding control. **b** Total RNA was purified and was used for qRT-PCR. Error bars represent means ± SD (**P < 0.01). **c** Exponentially growing cells were treated either with DMSO (control) or with cisplatin (20 and 30 μM) for 72 h. Cell viability was measured using the WST-1 assay. Error bars represent means ± SD (*P < 0.05).

Since AUF1 ectopic expression increased stem cell characteristics, we decided to determine whether the knockdown of AUF1 would reduce stemness. First, we evaluated the protein expression level of the stem cell markers CD44, CD24, and ALDH1 upon AUF1 knockdown in MDA-MB-231 cells. To this end, whole-cell lysates were prepared from MDA-AUF1si/MDA-C cells and were subjected to immunoblotting analysis. The obtained results revealed that AUF1 downregulation significantly reduced the level of CD44 and ALDH1, while increased the expression of CD24, suggesting that AUF1 downregulation inhibits stemness in these cells (Fig. 6a). The AUF1-dependent promotion of stemness was further confirmed by examining the effect of AUF1 downregulation on several pluripotent markers. Figure 6a shows that AUF1 downregulation reduced the protein level of the pluripotency markers KLF4, Oct-4, BMI1, and Sox2. This effect was confirmed at the mRNA level by showing that the downregulation of AUF1 in MDA-MB-231 cells significantly reduced the level of *KLF4*, *SOX2*, and *OCT-4* transcripts. These results indicate that AUF1 knockdown reduces stemness features in BC cells.

### AUF1 downregulation increases cisplatin sensitivity of BC cells

Since CSCs are known to play a key role in chemotherapy resistance and cancer recurrence, we sought to determine whether AUF1 downregulation would increase MDA-MB-231 cell sensitivity to the chemotherapeutic drug cisplatin. To this end, MDA-AUF1si/MDA-C cells were treated with cisplatin (20 and 30 μM) or DMSO (used as control) for 72 h, and then the WST-1 assay was performed to evaluate the cytotoxic effect. The obtained result indicates that MDA-AUF1si treated with 20 and 30 μM of cisplatin resulted in 33 and 11% increase in cell death compared to controls, respectively (Fig. 6c). These results indicate that AUF1 downregulation sensitizes BC cells to cisplatin.

## Discussion

In the present report we have clearly shown the role of AUF1 in inducing EMT in breast epithelial cells. Indeed, while ectopic expression of AUF1 upregulated the mesenchymal markers, downregulated the epithelial markers, and enhanced the migration/invasion and proliferation capacities of MCF10A and MCF7 cells, AUF1 downregulation in TNBC cells (MDA-MB-231 and BT-20) inhibited these EMT features. At the mechanistic level, we have shown that AUF1 upregulated *SNAIL1* and *TWIST1* through stabilizing their mRNAs. AUF1 binds the AU-rich elements and the potential AUF1-binding sites in the mRNA 3′UTR of both *SNAIL1* and *TWIST1*. This suggests that AUF1 promotes EMT in breast epithelial cells through post-transcriptional stabilization of these two major EMT-TFs. It has been previously shown that AUF1 stabilizes the EMT inducer ZEB1 in osteosarcoma and thyroid cancer cells^21,22^. Furthermore, other studies have reported the involvement of other RBPs in regulating EMT through stabilizing the mRNA of the three major EMT-TFs SNAIL1, TWIST1, and ZEB1. For instance, it has been shown that the RBP PTBP3 is an EMT inducer through stabilizing the *ZEB1* mRNA and promoting the migration/invasion and proliferation of BC cells^25^. Another mRNA stability-promoting protein is the human antigen R (HuR), which is overexpressed in many cancer types. HuR regulates hydrogen peroxide-induced SNAIL1 expression by stabilizing its mRNA, which subsequently enhances cell migration ability through suppressing E-cadherin expression^26^. In contrast, tristetraprolin (TTP) is a tumor suppressor RBP, which is deficient in several cancer types. Indeed, it has been shown that TTP negatively regulates EMT in ovarian cancer cells through binding the *SNAIL1* and *TWIST1* mRNAs and enhancing their degradation^27^. Together, these findings show that various RBPs are key players in cancer development and progression by post-transcriptionally regulating different EMT-TFs.

Recently, a link between EMT and CSCs has been established, and it became clear that EMT leads to the production of CSCs with stem cell characteristics such as self-renewal^7,8^. Furthermore, the ectopic expression of SNAIL1 or TWIST1 in human mammary epithelial cells induced EMT and also increased the ability of cells to form mammospheres and generate cells with CD44^high^/CD24^low^ characteristics^7^. In the present study, we have shown that the ectopic expression of p37^AUF1-ORF^ increases stemness features in MCF10A and MCF7 cells, indicating that AUF1 favors BCSC formation. This has been confirmed in vivo by showing that MCF7-ORF cells have higher ability to form tumors than their corresponding control cells. Indeed, MCF7-ORF xenografts had larger tumors compared to controls. On the other hand, AUF1 downregulation in MDA-MB-231 cells reduced the expression of stem cell markers CD44 and ALDH1, and upregulated CD24 at the protein level. Similarly, the Igf2 mRNA-binding protein 1 (IMP-1), another RBP protein, has been shown to induce loss in epithelial characteristics and acquisition of “stem-like” phenotype when overexpressed in colon cancer cells^28^. Indeed, IMP-1 downregulated E-cadherin indorses survival of single tumor cell-derived mammospheres and promoted a significant increase and maintenance of the CD44^high^/CD24^low^ population^28^. Together, these findings indicate that AUF1 and IMP-1 induce stemness features in non-carcinogenic and carcinogenic cells, and therefore they may play key roles in both the onset and the progression of tumors as well as in their resistance to therapy.

Cisplatin is a well-known platinum-based chemotherapeutic agent, widely used for the treatment of numerous cancers, including BC. Nonetheless, cisplatin has been associated with various side effects as well as drug resistance and tumor recurrence^29^. Growing evidence supports a critical role of CSCs in cancer chemotherapeutic resistance^30^. Intriguingly, several studies have shown that cisplatin induces CSC enrichment in various types of tumors^31,32^. Since AUF1 downregulation reduced the proportion of BCSCs, we hypothesized that this may sensitize BC cells to chemotherapy. Indeed, AUF1 knockdown by specific siRNA in MDA-MB-231 cells increased their sensitivity to cisplatin. This further confirmed the role of AUF1 in inducing stemness in BC, and also indicates the potential importance of targeting AUF1 to enhance the treatment of BC.

Together, these results show that the RNA-binding AUF1 protein plays important roles in breast carcinogenesis through stabilization of the key transcription factors TWIST1 and SNAIL1, and the consequent induction of EMT as well as stemness features. This indicates that, in the era of precision medicine, targeting AUF1 in BC cells could be of great therapeutic value for the hard-to-treat TNBC patients.

## Materials and methods

### Cells, cell culture, and reagents

MCF7, MCF10A, MDA-MB-231, and BT-20 cell lines were purchased from ATCC, and were cultured as recommended. Cell lines were authenticated using short tandem repeat profiling by ATCC, propagated, expanded, and frozen immediately into numerous aliquots after arrival. The revived cells were utilized within 10–12 passages and not exceeding a period of 3 months. Cells were regularly screened for mycoplasma contamination using MycoAlert Mycoplasma Detection Kits (Lonza, Basel, Switzerland). All supplements were obtained from Gibco. Cells were maintained at 37 °C in a humidified incubator with 5% CO_2_. Cisplatin was purchased from Sigma-Aldrich.

### Transfection and viral infection

*AUF1*-siRNA and control siRNA were obtained from Origene Technologies (Rockville, MD, USA). siRNA sequence: rGrCrCrArUrGrUrCrGrArArGrGrArArCrArArUrArUrCrAGC and universal sequence was used as a negative control. In addition, pSILENCER-AUF1-siRNA and control-siRNA plasmids were utilized^24^. The transfections were carried out using the High Perfect reagent (Qiagen), as recommended by the manufacturer. Cells were incubated for 3 days after transfection, recovered, and then were re-cultured for 3 days before collection for subsequent experiments. pLenti-GIII-CMV-hHNRNPD-GFP-2A-Puro (Expressing the p37^AUF1^ isoform) (Applied Biological Materials Inc.) and their control plasmids were used at 1 µg/ml each for transfection of 293FT cells. Lentiviral supernatants were collected 48 h post-transfection. Culture media were removed from the target cells and replaced with the lentiviral supernatant and incubated for 24 h in the presence of 1 µg/ml polybrene (Sigma-Aldrich). Transduced cells were selected after 48 h with puromycin. TWSIT1-shRNA and SNAIL1-shRNA and their control plasmids (Origen) were utilized to transfect MCF7-expressing AUF1-ORF using Lipofectamin 3000 following the manufacturer’s instructions (Invitrogen).

### Cellular lysate preparation and immunoblotting

This has been performed as previously described^33^. The list of the utilized antibodies is available in the Supplementary Experimental procedures. Each experiment was repeated twice.

### Quantification of protein expression level

Protein signal intensity of each band was determined using ImageQuant TL Software (GE Healthcare). Next, dividing the obtained value of each band by the value of the corresponding internal control allowed a correction of the loading differences. The fold change in the protein levels was determined by dividing the corrected values by that of the control. The levels of the phosphorylated proteins were normalized against the total amount of their relative non-phosphorylated forms.

### Determination of mRNA half-life

Cells were seeded in six-well plates, and were challenged the following day with Actinomycin D (5 µg/ml) for increasing periods of time. Total RNA was then extracted and subjected to qRT-PCR. The one-phase exponential decay curve analysis (GraphPad Prism) was used to assess the mRNA decay kinetics, considering the values at time 0 as 100%. The time corresponding to 50% remaining mRNA was considered as mRNA half-life. Experiments were performed three times.

### Dual-luciferase reporter assay

Cells were plated at 1 × 10^5^ cells/well on six-well plates and transfected with 3 µg of the luciferase/Renilla reporter vector containing either human full *TWSIT1* or *SNAIL1* 3′UTR, mutated sequence of the AUF1 seed sequence or a control sequence containing no-ARE sequence of *the* 3′UTR (GeneCopoeia). Transfection was carried out using Lipofectamin 2000 as recommended by the manufacturer (Invitrogen). At 24 h post-transfection, cells were seeded in 96-well plate and Firefly and Renilla luciferase activities were consecutively measured using the dual-luciferase assay as recommended by the manufacturer (GeneCopoeia). The Firefly luciferase signal was normalized to the Renilla luciferase signal for each individual analysis. The mean and SEM were calculated from three wells for each 3′UTR activity and presented as fold change over the non-stimulated control.

### Biotin pull-down analysis

The probes used to prepare biotinylated transcripts spanning the *TWIST* 3′UTR are: (wild type) ACUUGUCAACAAACACAAAAAAA and (mutated) ACUUGUCGCAGCACACAAAAAAA. *SNAIL-1* 3′UTR are: (wild type) AAGUUAUUUCGUCAAUUAAAUAUAUAA and (mutated) AAGUUAUUUCGUCGCCCGCAUAUAUAA. Biotinylation was performed using the RNA 3′ End Biotinylation kit as instructed by the manufacturer (Thermo Scientific, USA). Cytoplasmic lysates (200 µg per sample) were incubated with 3 µg of purified biotinylated transcripts for 30 min at room temperature, and then the complexes were precipitated with streptavidin-coupled Dynabeads (Invitrogen, USA) as previously described^34^. Proteins present in the pull-down material were analyzed by immunoblot analysis. Experiments were repeated twice.

### Cell proliferation, migration, and invasion assays

These assays were performed in a label-free real-time settings using the xCELLigence RTCA Technology (Roche, Germany) that measures impedance changes in a meshwork of interdigitated gold microelectrodes located at the well bottom (E-plate) or at the bottom side of a micro-porous membrane (CIM plate 16)^35,36^. Cell migration and invasion were assessed as per the manufacturer’s instructions. In brief, 2 × 10^4^ cells in serum-free medium were added to the upper wells of the CIM plate coated with a thin layer of Matrigel (BD Biosciences) basement membrane matrix diluted 1:20 in serum-free medium (invasion) or non-coated (migration). Complete medium was used as a chemo-attractant in the lower chambers. Subsequently, the plates were incubated in the RTCA for 24 h and the impedance value of each well was automatically monitored by the xCELLigence system and expressed as Cell Index (CI) value, which represents cell status based on the measured electrical impedance change divided by a background value. Experiments were performed three times in triplicate.

For the proliferation assay, exponentially growing cells (2 × 10^4^) were seeded in E-plate with complete medium as per the manufacturer’s instruction. Cell proliferation was assessed for 48 h. All data were recorded and analyzed by the RTCA software. Cell Index was used to measure the change in the electrical impedance divided by the background value, which represents cell status. Experiments were performed three times in triplicate^37^.

### Flow cytometry

Cells were stained with CD44/CD24 as previously described^38^. Briefly, cells were washed and incubated with CD44 APC-Cy7/CD24 Pacific Blue antibodies (both from Biolegend, USA) for surface staining (30 min at 4 °C).

### Spheroid formation

Cells were seeded in 96-well ultra-low attachment plate at a density of 1000 viable cells/well. Cells were cultured in 171 medium supplemented with 1% ABM, 2% B-27, 20 ng/ml EGF, 500 ng/ml HC, 4% FBS, and 5 μg/ml insulin. Cells were incubated for 10 days at 37 °C under 5% CO_2._ Mammospheres with a diameter of ≥50 μm were counted using an OPTIKA light microscope. Experiments were performed three times in triplicate.

### Soft agar colony formation assay

Details are available in Supplementary Experimental Procedures.

### Immunofluorescence

Details are available in Supplementary Experimental Procedures.

### Cytotoxicity assay

Cells 5 × 10^3^/well were seeded in 96-well plates with appropriate culture media. After cells treatment, WST-1 reagent (Sigma-Aldrich) was added to each well according to the manufacturer’s instructions. These experiments were performed in triplicate, and were repeated three times.

### Orthotopic tumor xenografts

Animal experiments were approved by the KFSH&RC institutional Animal Care and Use Committee (ACUC) and were conducted according to relevant national and international guidelines. Different cell concentrations of MCF7-ORF/MCF7-C cells (4 × 10^3^, 2 × 10^4^, 10^5^, 5 × 10^5^*n* = 3 for each cell concentration) were injected under the right and left nipples of 4 weeks female nude mice (NOD.CB17-Prkdcscid/J), respectively. Three animals for each cell concentration was estimated to be adequate for such experiment, and no randomization nor blinding was used. After 5 months, pictures of tumors were taken.

### Statistical analysis

Statistical analysis was performed by two-sided Student’s *t*-test and *P* values of ≤0.05 were considered as statistically significant.

## Supplementary information

Figure Legends Figure S1

Supplementary materials and Methods

Supplementary Figure 1
