# Supplementary material for: AUF1 promotes stemness in human mammary epithelial cells through stabilization of the EMT transcription factors TWIST1 and SNAIL1
Source: Oncogenesis. 2020 Aug 5;9(8):70. doi: 10.1038/s41389-020-00255-1 (PMC7406652; doi:10.1038/s41389-020-00255-1)
Supplement: Supplementary file 1 — Figure Legends Figure S1 [file 41389_2020_255_MOESM1_ESM.docx]

**Supplementary figure S1. AUF1 down-regulation inhibits the epithelial-to-mesenchymal transition process in breast cancer cells**

**A**, MDA-MB-231 cells were transfected with control or AUF1-siRNA (CTRL or AUF-1siRNA). Whole cell lysates were prepared and were used for immunoblotting analysis using antibodies against the indicated proteins, and GAPDH was used as internal control. **B***,* Total RNA was purified from the indicated cells and used for qRT-PCR. Experiments were performed in triplicate and several times; error bars represent means ± S.D (*P<0.05). **C**, Cell proliferation, migration and invasion abilities were assessed for the indicated periods of time using the RTCA-DP xCELLigence System. Data are representative of different experiments performed in triplicate.
