# Supplementary material for: AUF1 promotes stemness in human mammary epithelial cells through stabilization of the EMT transcription factors TWIST1 and SNAIL1
Source: Oncogenesis. 2020 Aug 5;9(8):70. doi: 10.1038/s41389-020-00255-1 (PMC7406652; doi:10.1038/s41389-020-00255-1)
Supplement: Supplementary file 2 — Supplementary materials and Methods [file 41389_2020_255_MOESM2_ESM.docx]

## **List of used antibodies**

## **RNA purification and qRT-PCR**

Quantitative RT-PCR was performed in triplicate using 4 µl cDNA mixed with 5 µl 2x FastStart Essential DNA Green qPCR mastermix (Roche, New York, NY, US) and 1 µl of 0.3 µM forward and reverse primers. Amplifications were performed utilizing the LightCycler 96 Real-time PCR detection system (Roche) using the following cycle conditions: 95°C for 10 min (1 cycle); 95°C for 10 sec, 59°C for 20 sec, 72°C for 30 sec (45 cycles). GAPDH expression levels were used for normalization, and gene expression differences were calculated using the threshold cycle (Ct). The used primers are as follow:

| Primers | Sequences | |
| --- | --- | --- |
| *AUF-1* | *Forward* | *5’-GAT CAA GGG GTT TTG GCT TT-3’* |
|  | *Reverse* | *5’-GTT GTC CAT GGG GAC CTC TA-3’* |
| *TWIST1* | *Forward* | *5’-GGA CAA GCT GAG CAA GAT TCA GA-3’* |
|  | *Reverse* | *5’-GTG AGC CAC ATA GCT GCA G-3’* |
| *SNAIL1* | *Forward* | *5’-CCT CAA GAT GCA CAT CCG AAG-3’* |
|  | *Reverse* | *5’-ACA TGG CCT TGT AGC AGC CA-3’* |
| *ZEB1* | *Forward* | *5’ GGC AGA GAA TGA GGG AGA AG-3’* |
|  | *Reverse* | *5’CTT CAG ACA CTT GCT CAC TAC TC-3’* |
| *GAPDH* | *Forward* | *5’-GAGTCCACTGGCGTCTTC-3’* |
|  | *Reverse* | *5’-GGGGTGCTAAGCAGTTGGT-3’* |
| *KLF4* | *Forward* | *5’CAT CTC AAG GCA CAC CTG CGA-3’* |
|  | *Reverse* | *5’-TCG GTC GCA TTT TTG GCA CTG G-3’* |
| *OCT4* | *Forward* | *5’CCT GAA GCA GAA GAG GAT CAC C-3’* |
|  | *Reverse* | *5’AAA GCG GCA GAT GGT CGT TTG G-3’* |
| *SOX2* | *Forward* | *5’GCT ACA GCA TGA TGC AGG ACC A-‘3* |
|  | *Reverse* | *5’TCT GCG AGC TGG TCA TGG AGT T-3’* |
| *c-MYC* | *Forward* | *5’GGA AGA AAT TCG AGC TGC-3’* |
|  | *Reverse* | *5’GCT GTC GTT GAG AGG GTA-3’* |
| *CD24* | *Forward* | *5’-CAC GCA GAT TTA TTC CAG TGA AAC-3’* |
|  | *Reverse* | *5’-GAC CAC GAA GAG ACT GGC TGT T-3’* |
| *CD44* | *Forward* | *5’-CCA GAA GGA ACA GTG GTT TGG C-3’* |
|  | *Reverse* | *5’-ACT GTC CTC TGG GCT TGG TGT T-3’* |
| *ALDH1A2* | *Forward* | *5’-GAG TAA CTC TGG AAC TTG GAG GC-3’* |
|  | *Reverse* | *5’-ATG GAC TCC TCC ACG AAG ATG C-3’* |
| *CDH1* | *Forward* | *5’-CCA GAA ACG GAG GCC TGA T-3’* |
|  | *Reverse* | *5’-CTG GGA CTC CAC CTA CAG AAA GTT-3’* |
| *CDH2* | *Forward* | *5’-CCT CCA GAG TTT ACT GCC ATG AC-3’* |
|  | *Reverse* | *5’-GTA GGA TCT CCG CCA CTG ATT C-3’* |

**Soft agar colony formation assay**

Cells were harvested and washed with serum free medium and 4x10^4^ cells were suspended in 4ml of defined medium with 0.3% agarose (A9045, Sigma, MD, USA). The mixture of agarose and cells was plated in 6-well plate containing a base layer of agarose of 0.5% (v/v). Cultures were incubated at 37 ^o^C in a humidified incubator. Plates were examined with an inverted microscope to confirm only single cells were plated without any clumps. Spent medium was changed with fresh medium every 3 days and cultures were continued for 21 days. Colonies with more than 100 μm were counted 2 weeks after initial plating. Experiments were performed three times.

**Immunofluorescence**

Cells and mammospheres were fixed with 4% paraformaldehyde for 10 min, permeabilized with 0.2% Triton X-100 for 10 min and quenched in 100 mM glycin for 10 min at room temperature. Cells were then blocked in 10% FCS and incubated with primary antibody. Cells were then washed and incubated with the Alexa Fluor-594 or -488 conjugated antibodies, respectively. Nuclei were stained with DAPI and Images were captured using Floid Cell Imaging Station (Life technologies).
