# Supplementary figures and images for: AUF1 promotes stemness in human mammary epithelial cells through stabilization of the EMT transcription factors TWIST1 and SNAIL1
Source: Oncogenesis. 2020 Aug 5;9(8):70. doi: 10.1038/s41389-020-00255-1 (PMC7406652; doi:10.1038/s41389-020-00255-1)

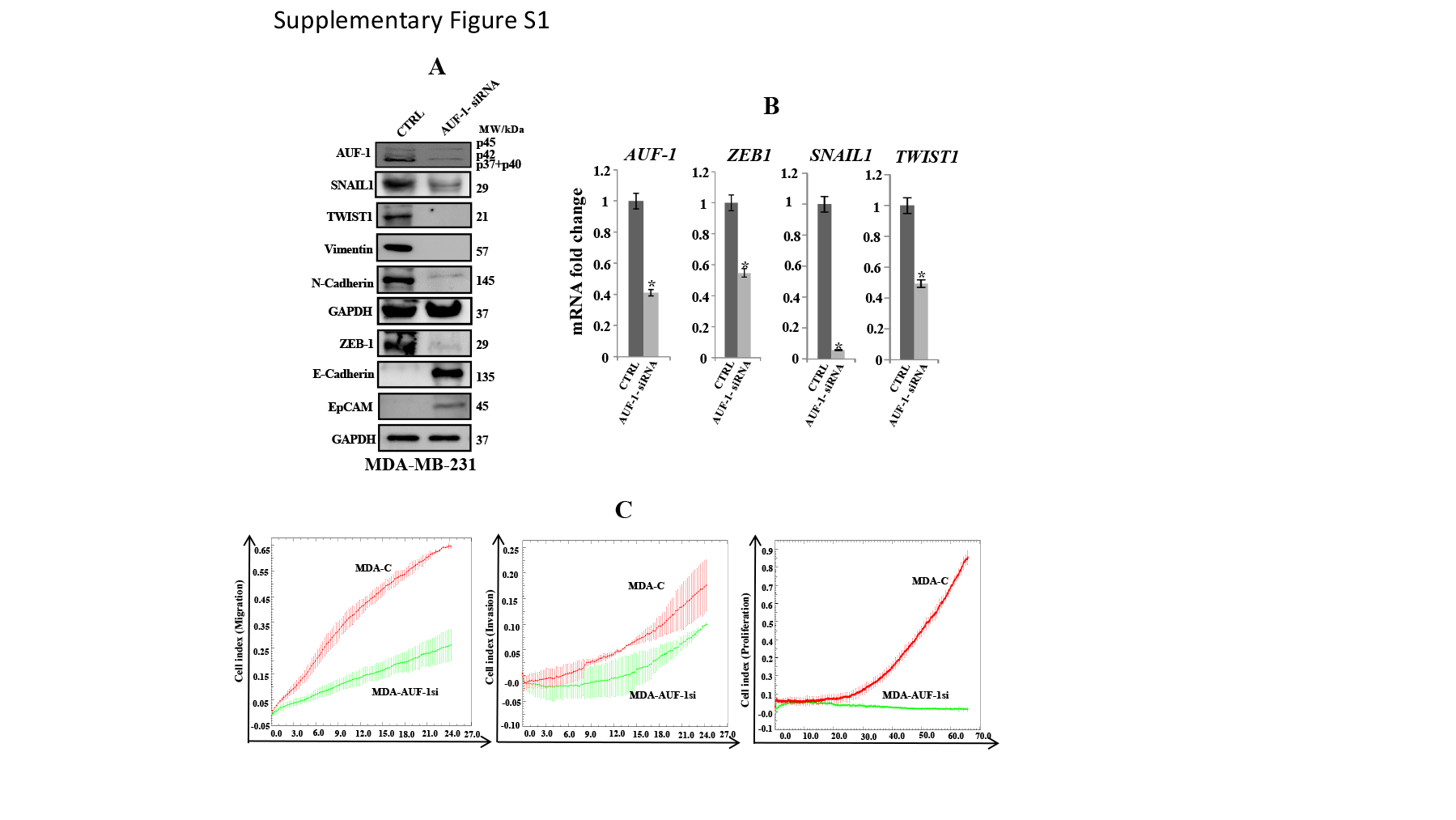

Supplement: Supplementary file 3 — Supplementary Figure 1 [file 41389_2020_255_MOESM3_ESM.tif]
